# Machine Learning Prediction of Hypoglycemia and Hyperglycemia From Electronic Health Records: Algorithm Development and Validation

**DOI:** 10.2196/36176

**Published:** 2022-07-18

**Authors:** Harald Witte, Christos Nakas, Lia Bally, Alexander Benedikt Leichtle

**Affiliations:** 1 University Institute of Clinical Chemistry Inselspital - Bern University Hospital and University of Bern Bern Switzerland; 2 Laboratory of Biometry University of Thessaly Volos Greece; 3 Department of Diabetes, Endocrinology, Nutritional Medicine and Metabolism Inselspital - Bern University Hospital and University of Bern Bern Switzerland; 4 Center of Artificial Intelligence in Medicine University of Bern Bern Switzerland

**Keywords:** diabetes, blood glucose decompensation, multiclass prediction model, dysglycemia, hyperglycemia, hypoglycemia

## Abstract

**Background:**

Acute blood glucose (BG) decompensations (hypoglycemia and hyperglycemia) represent a frequent and significant risk for inpatients and adversely affect patient outcomes and safety. The increasing need for BG management in inpatients poses a high demand on clinical staff and health care systems in addition.

**Objective:**

This study aimed to generate a broadly applicable multiclass classification model for predicting BG decompensation events from patients’ electronic health records to indicate where adjustments in patient monitoring and therapeutic interventions are required. This should allow for taking proactive measures before BG levels are derailed.

**Methods:**

A retrospective cohort study was conducted on patients who were hospitalized at a tertiary hospital in Bern, Switzerland. Using patient details and routine data from electronic health records, a multiclass prediction model for BG decompensation events (<3.9 mmol/L [hypoglycemia] or >10, >13.9, or >16.7 mmol/L [representing different degrees of hyperglycemia]) was generated based on a second-level ensemble of gradient-boosted binary trees.

**Results:**

A total of 63,579 hospital admissions of 38,250 patients were included in this study. The multiclass prediction model reached specificities of 93.7%, 98.9%, and 93.9% and sensitivities of 67.1%, 59%, and 63.6% for the main categories of interest, which were nondecompensated cases, hypoglycemia, or hyperglycemia, respectively. The median prediction horizon was 7 hours and 4 hours for hypoglycemia and hyperglycemia, respectively.

**Conclusions:**

Electronic health records have the potential to reliably predict all types of BG decompensation. Readily available patient details and routine laboratory data can support the decisions for proactive interventions and thus help to reduce the detrimental health effects of hypoglycemia and hyperglycemia.

## Introduction

### Blood Glucose Decompensations Are Associated With Poor Outcomes in Inpatients

Diabetes is one of the most common lifestyle diseases worldwide (affecting approximately 537 million people in 2021), particularly among older adults (aged >65 years), with numbers on the rise [1,2]. Consequently, the number of inpatients with diabetes is also increasing, with currently approximately every sixth hospital bed being used by patients with diabetes [3,4].

The hallmark of diabetes is loss of control over blood glucose (BG) levels. Failure to maintain BG levels within the ranges normally set by functional glucose homeostasis in nondiabetic people manifests as hyper- and hypoglycemia, where BG levels decompensate, that is, exceed or fall below a critical threshold, respectively.

Both hypoglycemia and hyperglycemia have been associated with numerous complications in inpatients. This includes an increased length of stay [5], diabetic ketoacidosis or hyperglycemic hyperosmolar state in the case of hyperglycemia [6], increased risk of infection [7,8], admission to intensive care units (ICUs) [9], and an overall increase in mortality [10-12]. The association between hypoglycemia and hyperglycemia and poor outcomes in inpatients who are critically and noncritically ill calls for a rigorous inpatient management approach toward reducing dysglycemia [13,14].

### Challenges of Inpatient Dysglycemia Management

The environment of inpatients (ie, a hospital setting) is usually well-controlled; nevertheless, the maintenance of BG levels in a normoglycemic range is demanding. It is complicated by the fragile health status of the inpatient, stress (including postoperative stress), prolonged fasting, changes in diet and meal timings, and inadequate dosing of or changes in the type of antidiabetic drugs administered to name a few main troubles [14,15]. Standard diabetes therapy using adjusted subcutaneous insulin injections (sliding-scale insulin) combined with insufficient glucose monitoring is another issue and a potential risk factor for hypoglycemia [16], aggravated by the frequent shortage of nursing staff in hospitals.

The combination of continuous glucose monitoring (CGM) systems and subcutaneous insulin pumps with automated insulin delivery (closed-loop) systems is a recent promising development [17-19], which may reduce the workload of clinical staff in addition to benefiting patients.

### Prediction of BG Decompensation

An alternative approach to continuous BG measurements is the prevention of BG decompensation by the detection of early signs or patterns associated with it, thereby avoiding the immediate and long-term adverse effects of hypoglycemia and hyperglycemia [20]. To date, studies have primarily assessed the prediction of hypoglycemia [20] based on, for instance, laboratory data [21,22] or data from electrocardiograms [23], or they required subcutaneous glucose readings from CGM systems [24].

### Aim of This Study

This study investigated whether readily available standard laboratory results and patient information could be used to reliably predict both hypoglycemia and hyperglycemia in inpatients with a clinically relevant prediction horizon.

## Methods

### Patient Cohort

The data set contained anonymized hospital admission data collected during the routine management of adult (aged ≥18 years) inpatients of the 6 hospitals of the Insel-Gruppe (Bern, Switzerland) from January 1, 2014, to December 5, 2019. Data were retrieved as is; that is, no extra data were collected for this retrospective cohort study. Data of patients whose BG levels had been assessed at least once were included if they met ≥1 of the following inclusion criteria:

Diagnosis of diabetes or diabetes-related syndromes (codes as specified in the 10th revision of the International Statistical Classification of Diseases and Related Health Problems of the World Health Organization [25]), including E10 to E14, E16, E66 to E68, G59, G63, H28, H36, K77.8, K85, M14.2, N08.3, O24, R73, and R81

Administration of an antidiabetic drug falling into code category A10 (or subcategories thereof) of the Anatomical Therapeutic Chemical Classification System

Extreme BG levels regardless of any formal diagnosis of diabetes, including a BG level of <4.0 or ≥11.1 mmol/L measured at any time, a fasting venous BG level of ≥7.0 mmol/L, a 2-hour value of ≥11.1 mmol/L during an oral glucose tolerance test, or an HbA_1c_ value of ≥48.0 mmol/mol (International Federation of Clinical Chemistry) or ≥6.5% (Diabetes Control and Complications Trial and National Glycohemoglobin Standardization Program)

The rationale behind these 3 inclusion criteria was to include a broad range of patients with potential indications of dysglycemia. The fact that BG level tests belong to the routine panel of laboratory tests in inpatients further helped reduce potential cohort bias. The unfiltered inclusion of all patients’ BG measurement data was not possible because of the limitations set by the Swiss Human Research Act.

In total, the patient cohort comprised 38,250 patients (n=16,842, 44.03% women and n=21,408, 55.97% men) who had undergone 63,579 hospital admissions (cases), during which at least 1 laboratory analysis of 52 parameters was performed (Multimedia Appendix 1).

### Definition of Dysglycemia

The types of BG decompensation are defined by BG levels of <3.9 mmol/L (<70 mg/dL; hypoglycemia) [26] or >10, >13.9, or >16.7 mmol/L (representing different degrees of hyperglycemia; >180, >250, and >300 mg/dL, respectively) [26,27]. A second level of hypoglycemia (BG <3.0 mmol/L [<54 mg/dL]) [26] was not considered specifically as both BG levels of <3.0 or <3.9 mmol/L may lead to an “altered mental and/or physical status requiring assistance” [26]; that is, a BG level that is <3.9 mmol/L may already pose a significant risk for a patient.

BG level intervals were assigned category numbers of 0 to 4, with category 0 representing cases not showing BG decompensation (Table 1). These categories were selected on the basis of clinical relevance, intuition in interpretation, and clinicians’ decision-making and were aligned with clinical practice guidelines [26,27].

**Table 1:** Category assignment of blood glucose decompensation types.

Blood glucose level interval (mmol/L)	Category	Decompensation type
≥3.9 to ≤10	0	Nondecompensated
<3.9	1	Hypoglycemia (level 1)
>10 to ≤13.9	2	Mild hyperglycemia
>13.9 to ≤16.7	3	Moderate hyperglycemia
>16.7	4	Severe hyperglycemia

### Data Preprocessing

Before use, the data sets were cleaned and preprocessed to remove erroneous entries and unreasonable, unlikely, or even impossible values.

For patient variables, values outside the following (reasonable) limits were set to “N/A” (whereas all other values of the respective patient were retained) as they are most likely the result of errors during data input: ages 18 to 130 years, body height 100 to 250 cm, and weight 25 to 400 kg. For laboratory measurements, negative values were removed (apart from the measurements of base excess, which allowed negative results). Values generally *incompatible with human life* were published only for a relatively small number of analytes [28], and measurements outside the following limits were excluded on the basis of such published limits for chloride (<65 or >138 mmol/L) [29], plasma pH (<6.8 or >7.8) [30,31], potassium (<1.3 or >9.0 mmol/L) [29], and sodium (<100 or >191 mmol/L) [29].

Other putative outliers were identified using the *Isolation Forest* algorithm [32] (implemented in the Python *sklearn.ensemble. IsolationForest* module; version 0.21.3) and were flagged. These binary outlier flags were used as additional variables to label potentially severe cases.

### Predictors and Outcomes

**Figure 1:**
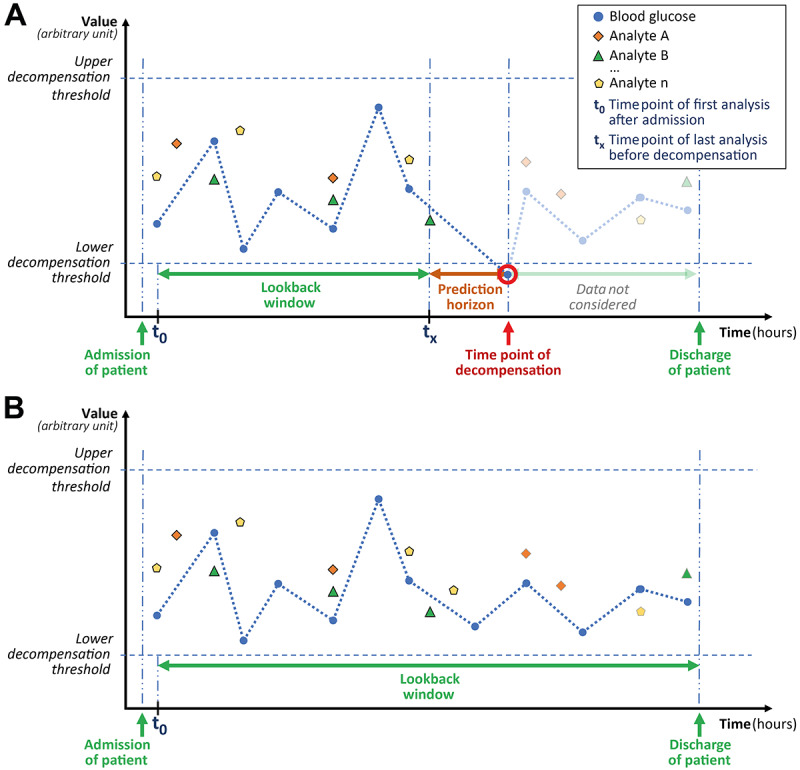
Look-back window and prediction horizon (A: decompensated case; B: nondecompensated case).

Decompensation events corresponding to the different decompensation categories specified in Table 1 were identified in the data for each admission case. If an event corresponding to a decompensation category was present, all data collected before the decompensation event (look-back window; see Figure 1 for a fictive case with [Figure 1A] and without [Figure 1B] a decompensation event) were used to form derived variables for each laboratory analyte (descriptive statistics: mean, SD, IQR, total range, recent trend, most extreme and most recent value, and analysis count). These derived variables are supposed to reflect the current overall status of a patient, mimicking a physician’s assessment of the information present in a patient’s health record. Only the first decompensation event was considered for each category. If a decompensation event was detected with the first measurements of a case, it was not considered at all in the corresponding decompensation categories. If no decompensation event of a specific category occurred within a case, the data collected at all its time points were used to create the derived variables (Figure 1B). The time span from the last piece of data of at least 1 predictor variable in the electronic health record (EHR) before the decompensation event of interest was considered the prediction horizon for the respective category (Figure 1A).

Analyte statistics were subsequently combined with patient demographics (age, sex, weight, height, language, and civil status), information on the previous and current administration of antidiabetic drugs, previous incidents of BG decompensation, history of diagnoses, and stay in ICU (complete variable list is provided in Multimedia Appendix 2). The data points were hashed, duplicates were removed, and a final data table was generated for the development and evaluation of the prediction models.

### Prediction Models and Second-Level Ensemble

**Figure 2:**
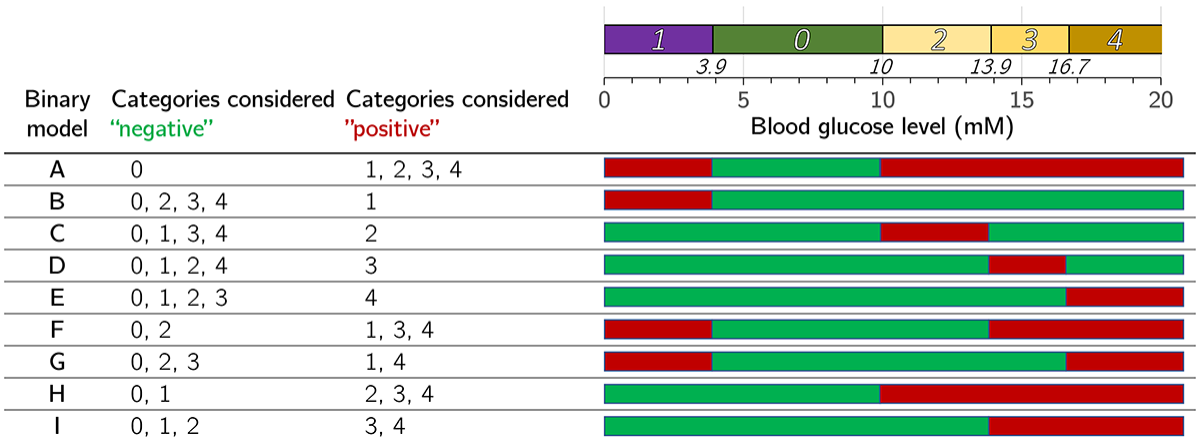
Binary models detecting clinically relevant combinations of blood glucose decompensation types.

Binary prediction models were set up to distinguish clinically relevant combinations of decompensation categories (Figure 2). To this end, decision tree–based classification models using extreme gradient boosting (XGBoost; XGBoost package [33]; version 1.0.0.2) were trained. XGBoost models do not make any assumptions about the distribution of the data and can deal with incomplete data sets. This is particularly useful for clinical data where the time points and types of analyses are specific for each individual patient. XGBoost uses a sparsity-aware split-finding algorithm that assigns a default direction to each node [33]. If a feature value is missing, the default direction along the corresponding node is taken [33,34].

The data of cases falling into several categories were used to train each of the relevant prediction models. For each binary model, cases that did not meet the respective decompensation criterion were used as controls. The resulting categorizations may be puzzling for the human observer at first glance; for example, a binary hypoglycemia model using the data of a hyperglycemic case as *control*.

The model parameters were optimized over a wide parameter space using a stochastic grid search approach covering 2000 to 4000 parameter combinations. Data were split 70/30; that is, 70% of the cases were used for iterative training and the withheld 30% for model testing.

To counter the imbalance in the data sets, each sample was assigned a weight corresponding to the inverse frequency of its class during model training.

The best models with respect to precision (positive predictive value), sensitivity (recall), area under the curve of the receiver operating characteristic curve (AUC ROC), and informedness were retained. The *importance* of each variable (feature importance), which is a score indicating how influential each variable was during the construction of the tree ensemble, was determined during model training [33]. The selection of variables in advance was not required for the XGBoost models. Feature importance was calculated for each separate decision tree and node as the amount by which splitting on that particular node reduces the loss function, weighted by the number of leaf assignments to which the node contributes. Therefore, features affecting more decisions and decisions with higher significance will have higher relative importance. For the final assignment, the importance was averaged across the entire ensemble for each variable [33,35].

Subsequently, multiple binary models were assembled into a second-level ensemble to build a multiclass classifier. The composition of this second-level ensemble was optimized for average precision using a genetic algorithm [36,37], as the number of possibilities for combining binary models into an ensemble grows exponentially.

Each multiclass is described by a (theoretical) combination of predictions of all binary classifiers (class-specific bit strings, ie, *ideal results*). For multiclass classification, a case-specific bit string for the votes of all binary classifiers of the second-level ensemble was created. The label of the *ideal* class-specific bit string with the minimal Hamming distance to the predicted case-specific bit string was chosen as the predicted case label.

In the case of a tie between ≥2 classes, they were prioritized according to clinical relevance (severity): hypoglycemia>severe hyperglycemia>moderate hyperglycemia>nondecompensated case>mild hyperglycemia. Mild hyperglycemia was assigned the lowest priority to reduce false alarms.

### Ethics Approval

This study was approved by the Bernese cantonal ethics committee (Kantonale Ethikkommission, KEK) and registered with the Business Administration System for Ethics Committees of the canton of Bern (KEK/NZE file number: Req-2018-00335).

## Results

### Patient Cohort Characteristics

For this study, we analyzed clinical data from 38,250 inpatients who had undergone 63,579 hospital admissions between January 1, 2014, and December 5, 2019, during which at least 1 laboratory analysis was performed. Of the 38,250 patients, 16,842 (44.03%) were women (age: mean 62.6, SD 19.6 years), and 21,408 (55.97%) were men (age: mean 65.9, SD 14.4 years). Specific details for the different categories of dysglycemia (based on clinical relevance; compare with the *Methods* section) are summarized in Table 2.

**Table 2:** Patient cohort characteristics at baseline.

		Category				
		0: nondecompensated	1: hypoglycemia	2: mild hyperglycemia	3: moderate hyperglycemia	4: severe hyperglycemia
**Characteristics**						
	Number of cases^a^	24,330	8164	26,788	10,419	8484
	Prevalence per admission, %	38.3	12.8	42.1	16.4	13.3
**Gender, n (%)**						
	Female	10,820 (44.5)	3911 (47.9)	9981 (36.9)	3687 (35.4)	2989 (35.2)
	Male	13,510 (55.5)	4253 (52.1)	16,897 63.1)	6732 (64.6)	5495 (64.8)
Age (years), median (IQR)		69 (56-78)	65 (48-75)	69 (60-77)	68 (60-77)	68 (58-76)
Height (cm), median (IQR)		169 (163-175)	168 (162-175)	170 (163-176)	170 (163-176)	170 (163-176)
Weight (kg), median (IQR)		81.9 (69.5-96.5)	71.0 (60.3-83.4)	79.1 (67.9-91.9)	79.6 (68.1-92.4)	78.8 (67.0-91.0)
BMI, median (IQR)		28.4 (24.7-33.4)	25.0 (21.7-29.0)	27.3 (24.0-31.4)	27.5 (24.1-31.7)	27.3 (23.8-31.5)
Cases in the ICU^b^, n (%)		1381 (5.7)	2062 (25.3)	7694 (28.7)	3171 (30.4)	2523 (29.7)
Length of stay (days), median (IQR)		3.8 (1.9-7.6)	7.6 (3.6-14.8)	6.8 (3.0-12.1)	7.1 (3.2-13.1)	6.9 (3.1-13.1)
Previous diagnosis of diabetes, n (%)		9529 (39.2)	2449 (30)	9860 (36.8)	4568 (43.8)	3985 (47)
Antidiabetic drugs (administered before hospital admission), n (%)		8046 (33.1)	1975 (24.2)	8396 (31.3)	3829 (36.8)	3291 (38.8)
Previous BG^c^ decompensation, n (%)		6801 (28)	4238 (51.9)	10,820 (40.4)	7885 (75.7)	6415 (75.6)
Decompensation level (mM), median (IQR)		N/A^d^	3.6 (3.2-3.7)	11.4 (10.6-12.3)	14.9 (14.4-15.7)	18.6 (17.4-20.8)
Time point of decompensation (hours; time after hospital admission), median (IQR)		N/A	34 (4-115)	16 (1-43)	25 (3-70)	22 (1-74)

^a^Numbers sum up beyond the total number of hospital admissions (63,579), as cases occasionally fell into multiple categories if different decompensation events occurred within a case (Figure 3).

^b^ICU: intensive care unit.

^c^BG: blood glucose.

^d^N/A: not applicable.

Men represented approximately two-thirds of the patients showing a hyperglycemic decompensation event (Table 2), whereas nondecompensated and hypoglycemic cases mirrored the overall gender distribution of the cohort.

The patients showing hypoglycemia included significantly younger patients compared with other categories, and similarly, patients presenting with hypoglycemia were less obese (ANOVA [type II] using subjects as blocking factors, followed by the Games-Howell post hoc test 95% confidence level; *P*<.001). The likelihood that patients with some form of BG decompensation were admitted to the ICU increased approximately 5-fold.

An overview of the baseline characteristics of the patient cohort is provided in Table 2.

**Figure 3:**
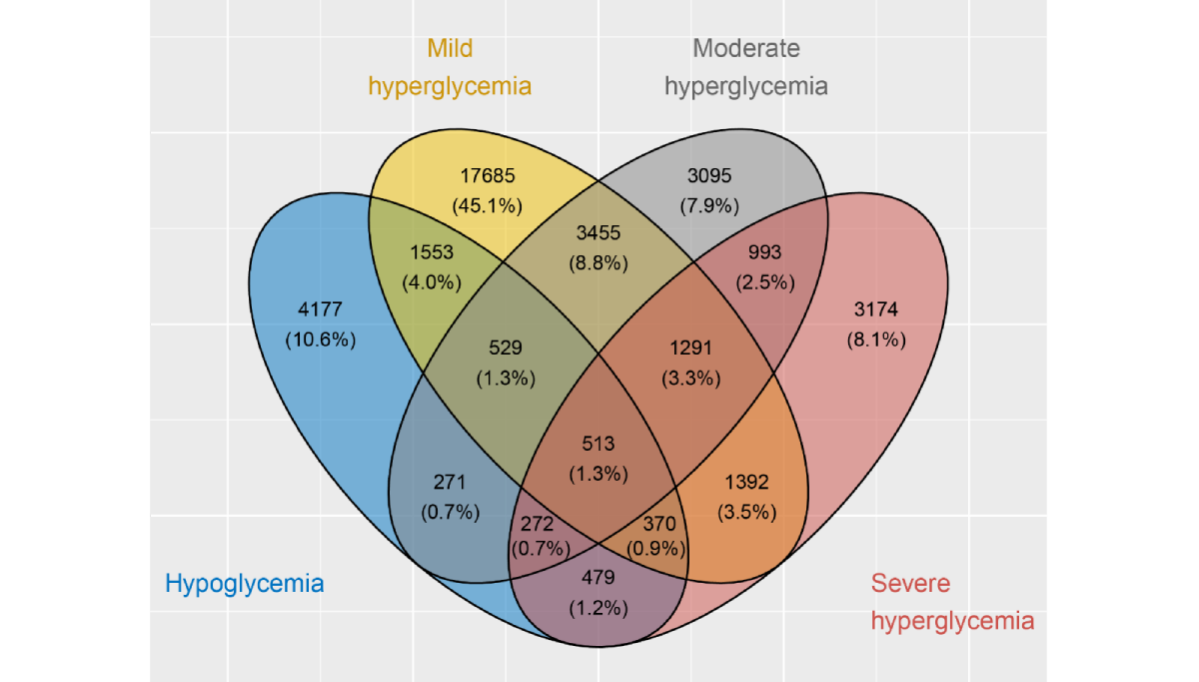
Types of decompensation events.

In a subset of cases, the patients showed multiple episodes or categories of decompensation events during admission (Figure 3). This included opposing decompensation types (hypo- and hyperglycemia) during the same admission in approximately 10.16% (3987/39,249) of decompensated cases. The inclusion criteria of the cohort favored a high prevalence of decompensation events (eg, patients with a formal diagnosis of diabetes or diabetes-associated comorbidities); hence, the prevalence of cases without any kind of BG decompensation was appreciably low (Table 2).

### Multiclass Classifier Performance

Several binary models were assembled into a second-level ensemble to create a multiclass classifier. For *k* classes, a minimum of *log_2_k* binary classifiers are required to represent all classes, (ie, a minimum of 3 classifiers for 5 classes). Adding additional binary classifiers (beyond *log_2_k*) is, in principle, redundant; however, additional bits can act as error-correcting codes (eg, the study by Berger [38]).

As the number of potential ensembles built from binary models grows exponentially, their composition was optimized using a genetic algorithm [36,37], assessing different combinations of the best binary models with respect to precision, sensitivity (recall), AUC ROC, and informedness. Average precision served as a readout (*fitness value*) during the optimization process.

The multiclass classifier ensemble with the best performance comprised binary model types A, B, G, H, and I (compare Figure 2; ie, of models trained to recognize all types of decompensations, only hypoglycemia, hypoglycemia and severe hyperglycemia, all types of hyperglycemia, and moderate and severe hyperglycemia, respectively). The mean AUC ROCs of the contributing binary models were 0.925 (SD 0.001), 0.960 (SD 0.002), 0.867 (SD 0.002), 0.914 (SD 0.001), and 0.863 (SD 0.003), respectively (5-fold cross-validation).

**Figure 4:**
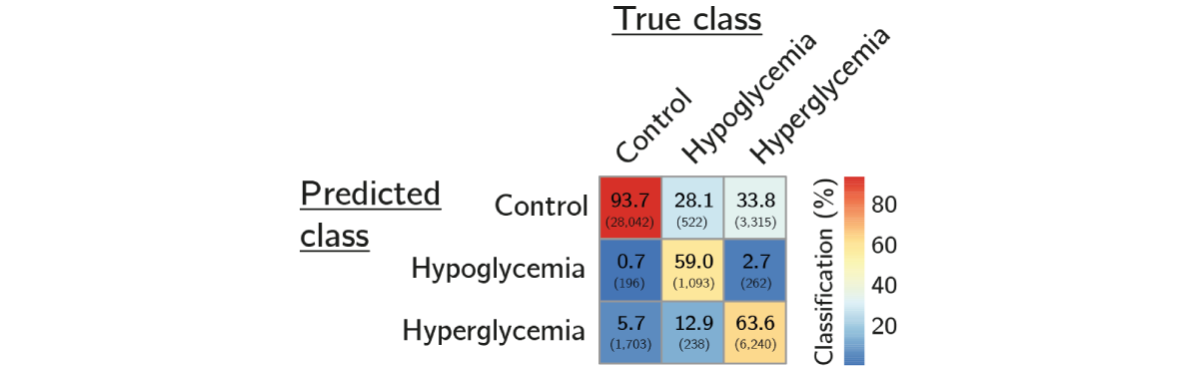
Classification by multiclass classifier for nondecompensated cases (control), hypoglycemia, and hyperglycemia (confusion matrix; percentages relative to true class; number of cases in parentheses).

An overview of the true decompensation categories versus the model predictions (confusion matrix) is shown in Figure 4. Our multiclass classifier correctly predicted nondecompensated, hypoglycemic, and hyperglycemic cases in 93.66% (28,042/29,941), 58.99% (1093/1853), and 63.56% (6240/9817) of cases, respectively, in relation to the true category.

The performance metrics for each class are summarized in Table 3. All types of dysglycemia were predicted reasonably but somewhat conservatively.

**Table 3:** Performance of multiclass classifier by class (5-fold cross-validation).

Performance metric	Performance (%), mean (SD)		
	Control	Hypoglycemia	Hyperglycemia
Sensitivity	67.1 (2.3)	59.0 (5.7)	63.6 (1.1)
Specificity	93.7 (0.9)	98.8 (0.5)	93.9 (0.6)
Precision	80.6 (1.9)	71.8 (8.6)	76.3 (1.6)
Balanced accuracy	80.4 (0.7)	78.9 (2.6)	78.7 (0.4)

**Figure 5:**
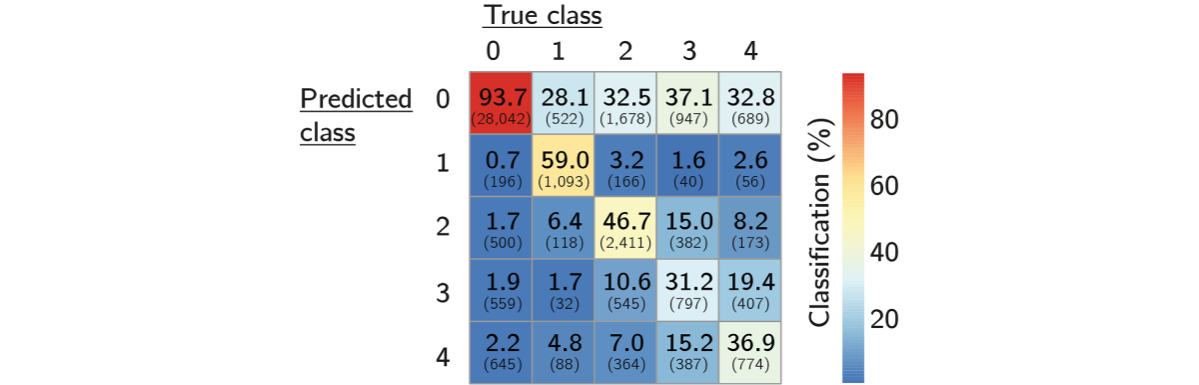
Classification by multiclass classifier (confusion matrix; percentages relative to true class; case numbers in parentheses).

When the different types of hyperglycemia were considered separately, mild, moderate, and severe hyperglycemia were correctly predicted in 46.69% (2411/5164), 31.22% (797/2553), and 36.87% (774/2099) of the cases in relation to the true category, respectively (Figure 5). For each type of hyperglycemia (mild, moderate, and severe), an additional 20% to 30% of the classifications fell into other categories of hyperglycemia, meaning that the overall state of hyperglycemia was detected in those cases as well.

Nondecompensated cases or cases showing hypoglycemia and severe hyperglycemia were predicted reasonably, albeit fairly conservatively, with lower sensitivity. The correct distinction between hyperglycemia types was more challenging. From a clinical perspective, this is understandable as these cases form a continuum. Nevertheless, a warning of any kind of hyperglycemia makes no difference in terms of the measures taken; hence, a classification of hyperglycemia was assumed to be sufficient, as long as this is indeed the underlying condition.

The median look-back window was 4.4 (IQR 1.8-9) days, 1.9 (IQR 0.4-5.7) days, 1.0 (IQR 0.3-2.4) days, 1.5 (IQR 0.6-3.7) days, and 1.8 (IQR 0.7-4.4) days for control class, hypoglycemia, and mild, moderate, and severe hyperglycemia, respectively. The median prediction horizon was 7 (IQR 3-15) hours for hypoglycemia and 4 (IQR 3-7, 3-6, and 3-6 for mild, moderate, and severe hyperglycemia, respectively) hours for all hyperglycemia types. In a clinical setting, such a prediction horizon would allow sufficient time for proactive interventions before BG decompensation occurs.

If the multiclass classifier is converted (reduced) to a binary classifier, it correctly predicts 67.12% (7833/11,670) of decompensated and 93.66% (28,042/29,941) of nondecompensated cases, corresponding to sensitivity (recall) and specificity (selectivity), respectively. In terms of precision (positive predictive value) and balanced accuracy, the model achieved 80.5% and 80.5%, respectively.

**Figure 6:**
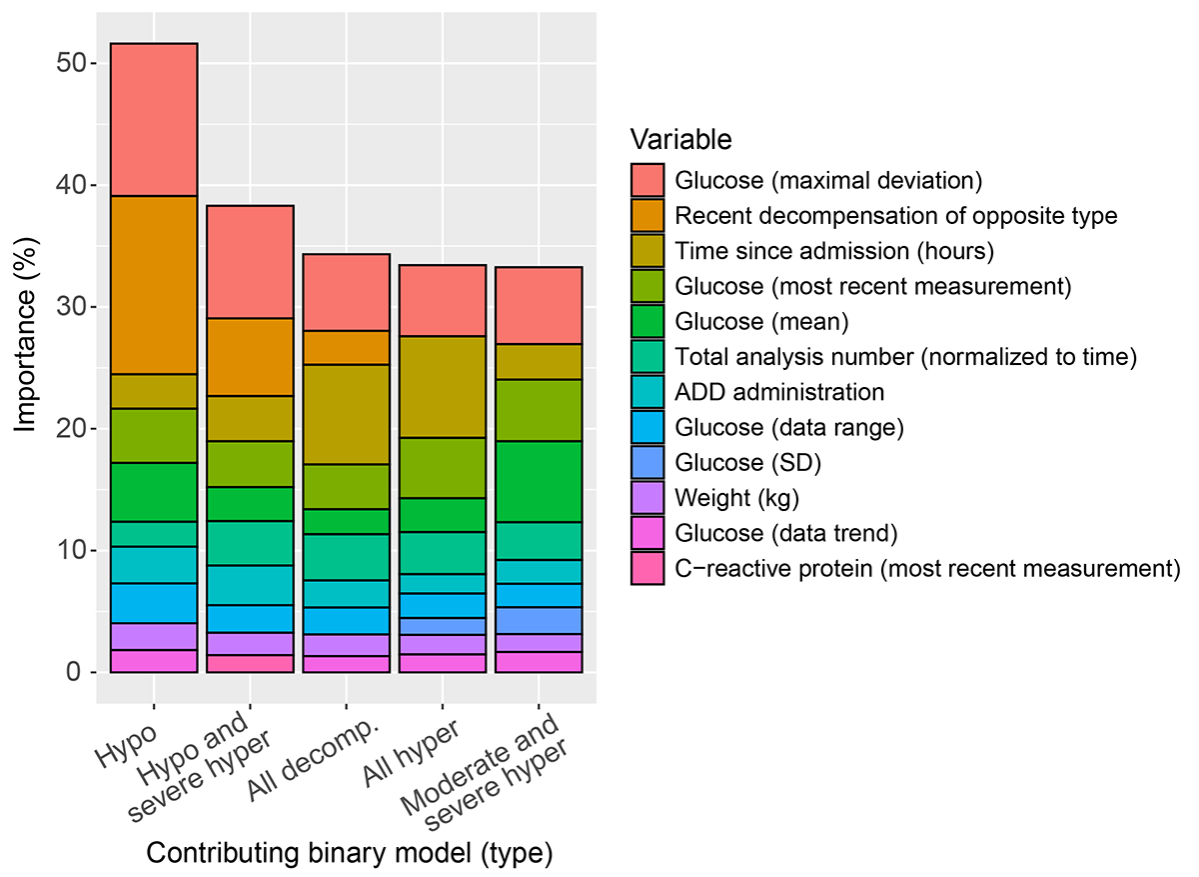
Variable importance (population level) of binary models comprising the multiclass classifier (top 10 shown). ADD: antidiabetic drug; all: all types of; decomp: decompensation; hyper: hyperglycemia; hypo: hypoglycemia.

Notably, across all predictions, a relatively small set of variables had a comparably large impact on the individual binary models constituting the multiclass classifier (Figure 6). Although the contributions of the variables (feature importance; see the *Methods* section for details) varied slightly across models, they all pointed in the same direction. A set of 12 variables encompassed the 10 most important variables for all 5 binary models. Not unexpectedly, quantitative readouts of glucose measurements had the highest importance; however, *soft* variables, such as information on recent decompensation events or time since admission, were also critical.

## Discussion

### Principal Findings

In this study, we set up a prediction model for hypo- and hyperglycemia from routine inpatient clinical data. We used anonymized EHRs of 63,579 hospital admissions of 38,250 patients between January 2014 and December 2019 to derive variables from measurements of common laboratory analytes, as well as patient information, drug administration, and diagnosis history. The data were characterized by a high degree of missingness, particularly for many of the variables originating from less common laboratory tests. To address this issue, we created a second-level ensemble comprising binary decision tree models that could deal with incomplete data. With respect to the overall main categories of interest (nondecompensated cases and hypo- or hyperglycemia), our classifier achieved 93.7% (28,042/29,941), 59% (1093/1853), and 63.6% (6240/9817) correct predictions, respectively. When converted to a binary classifier, it reached correct classification rates of 93.7% (28,042/29,941) and 67.1% (7833/11,670) for decompensated and nondecompensated cases, respectively. The median prediction horizon was 7 and 4 hours for hypo- and hyperglycemia, respectively.

### Strengths and Limitations

In our modeling approach, the variables were derived rather than directly taken from the EHRs. This increases the effort in an applied clinical setting as the variables need to be updated when new measurements are added to a patient’s EHR. This concerns both the summary statistics of each analyte and variables related to patient history (eg, application of antidiabetic drugs or previous decompensation events), which may change during hospitalization. However, an automatic update from clinical data warehouses should solve this issue. The ultimate aim should be to allow integration into a real-time alert system.

Data were not specifically collected for our retrospective study; hence, our data set may show a potential bias in terms of the timing of blood sampling; for example, hours during the day with higher staffing levels or before meals may be somewhat overrepresented. Hypoglycemia, in particular, may occur at night or in the early morning hours when no measurements are performed. Despite this potential bias in the training data, our model featured a promising prediction horizon. With 7 and 4 hours for hypo- and hyperglycemia, respectively, it provided sufficient time to initiate measures and prevent BG decompensation and its adverse effects. The difference between hypo- and hyperglycemia may be explained by more deliberate monitoring of BG levels in potential hypoglycemic patients by clinical staff.

The main strength of our model’s performance lies in the distinction between the extreme ends of the decompensation scale (nondecompensated cases, hypoglycemia, and severe hyperglycemia). However, the discrimination rate of different types of hyperglycemia, was rather moderate, particularly the rate of correct classification of moderate hyperglycemia (31.2%). In part, this may be because the categorization of different types of hyperglycemia, and the overall distinction into 5 categories in our model, had a clinical relevance rather than a computational one. Different types of hyperglycemia are defined by clinical practice guidelines [26,27] and form a continuum in the daily routine.

This does not affect the model’s usefulness drastically when taking into account the intention behind a model like ours, namely serving as decision support for adjustments of patient monitoring or therapeutic strategies (not triggering any treatment such as administration of insulin or glucose). Regarding the potential consequences of misclassification for a patient, mix-ups of hyperglycemia types are clinically irrelevant, and similarly, confusion of hypoglycemia and hyperglycemia has no drastic consequences—they would all *trigger* a BG test by clinical staff. False positives cause an extra workload for clinical staff but are also noncritical for a patient and occurred at a low rate (6.3%). False-negative predictions are more problematic as they may possibly result in the noninitiation of a BG test or countermeasures for BG decompensation. The false-negative rate of our model (32.9%) is suboptimal, meaning that it classifies too conservatively and misses too many decompensated cases. However, in light of these cases being missed completely without decision support, this is a step in the right direction.

Despite having a common denominator—diabetes or diabetes-related comorbidities—patients in our cohort were diagnosed with various primary and secondary diagnoses. Consequently, a plethora of different analyses have been conducted, with some tests done routinely; for example, the assessment of blood count, and other, more specific ones, were missing in most cases, such as analyses related to iron metabolism. Our prediction model, a second-level ensemble using derived variables, deals well with this sparse data (shown in the *Methods* section), in contrast to other popular methods such as logistic regression, support vector machines, and neural networks, which require complete data sets.

Akin to other studies [22], the patient cohort underlying our model features a certain bias toward patients with potential indications of dysglycemia (see the *Methods* section). However, this is not necessarily disadvantageous. A cohort without preselection may lead to trivial models calling for the (nondecompensated) group with an overwhelming frequency, yielding a low overall classification error but limited clinical utility [39]. The actual performance, clinical applicability, and performance for specific patient subgroups (eg, dysglycemia risk patients) must be assessed in broader follow-up studies, as for every model.

Notably, our model uses routine clinical data from standard laboratory tests and patient information, all of which are readily available in hospital settings. This is beneficial as these variables—assuming a proper in-house information flow—are available for free, both in terms of cost (no additional testing required) and effort (once the information is entered). However, such data collected during routine management usually do not feature fixed sampling times or a common set of analyses, which complicates alignment between cases. Given the absence of periodicity in the data, our approach was to mimic a physician’s intuitive assessment of the information present across a patient’s health record (eg, average and extreme values, trends, and most recent values) rather than individual data points.

This could ensure broad applicability, for example, in contrast to models based on expensive specialized tests [22] or even data from sensor implants [24]. Without a doubt, CGM devices offering frequent subcutaneous glucose measurements are ideal for a narrow set of patients, such as those with type 1 diabetes. However, their wider use in the general context of inpatients is neither practical nor cost-effective. In times of tight budgets for health care systems, the frugality of a model relying only on pre-existing data is of additional value. Our model does not require data sampled at specific time points; it takes whatever data are available. The identification of high-risk patients at no extra cost may lead to a reduction in workload for clinical staff and less frequent blood sampling for average patients. Overalerting should be taken into account as well—it would reduce the benefit of correct predictions; hence, the rather conservative nature of our multiclass classifier may actually not be such a drawback. A model is always an approximation of reality and should serve its purpose in the first place (“All models are wrong but some are useful.” [40]).

### Comparison With Prior Work

An increasing number of prediction studies are using the growing amount of patient data available in EHRs (for review, see the studies by Woldaregay et al [20], Roca et al [41], and Torkamani et al [42]). Diabetes is a popular and promising target of prediction studies [20] because of both its high prevalence in an aging population and the associated economic burden. The growing number of inpatients with deficiencies in controlling their BG levels calls for a refinement of the existing inpatient dysglycemia management [12-14,43].

Models for predicting a single type of BG decompensation as diabetes-associated complications have been established previously, mainly for hypoglycemia [20-22,44,45]. Few studies only have been published for hyperglycemic events [20].

To the best of our knowledge, this is the first general multiclass prediction model for BG decompensation to date (ie, both hypo- and hyperglycemia). A multiclass model offers the advantage of overcoming the vagueness associated with a binary model in a setting of >2 classes. For example, the prediction of *nonhypoglycemic* by a hypoglycemia model is ambiguous as the affected patient could be *nondecompensated* or *hyperglycemic*, where the latter would require action by the patient or clinical staff. A multiclass model such as ours resolves this conflict by differentiating between nondecompensated and multiple types of decompensated cases.

Notably, our model performed reasonably well when reverted to a binary model. Although it may be counterintuitive to first build a multiclass classifier and then revert it to a binary classifier, this approach takes advantage of the fact that ensembles tend to outperform individual models [46] and can benefit from error correction [38].

### Future Directions

In the next step, it would be interesting to assess the multiclass classifier in a follow-up retrospective cohort study for validation purposes using an independent data set. Optimizing the sensitivity and reducing the false-negative rate should be an additional focus to make the model more applicable for clinical use. Further down the road, the incorporation of the model into an alert system or even actionable artificial intelligence [17-19] could be tested. This would allow the evaluation of its real-time effectiveness, ideally leading to a reduction in the incidence of BG decompensations in inpatients.

### Conclusions

Our multiclass prediction model based on derived variables can classify both hypo- and hyperglycemia with reasonable sensitivity.

Given the serious adverse health effects of hypo- and hyperglycemia and the associated poor outcome of BG decompensation in inpatients, it is important to prevent dysglycemia whenever possible. Prediction models such as ours may support clinicians in inpatient management by proactively pointing out the necessity for adjustment of patient monitoring or therapeutic strategies. Therefore, this study may serve as a step toward a real-time alarm system and actionable artificial intelligence, which may aid in reducing BG decompensation in inpatients.

## Appendix

Multimedia Appendix 1 Descriptive statistics of laboratory measurements used in this study.

Multimedia Appendix 2 List of variables used for model setup.

## Abbreviations

AUC ROC: area under the curve of the receiver operating characteristic curve
BG: blood glucose
CGM: continuous glucose monitoring
EHR: electronic health record
ICU: intensive care unit
XGBoost: extreme gradient boosting
