# Supplementary material for: Machine Learning Prediction of Hypoglycemia and Hyperglycemia From Electronic Health Records: Algorithm Development and Validation
Source: JMIR Form Res. 2022 Jul 18;6(7):e36176. doi: 10.2196/36176 (PMC9345028; doi:10.2196/36176)
Supplement: Multimedia Appendix 1 [file formative_v6i7e36176_app1.pdf]

Multimedia Appendix 1

Witte *et al.*, 2022: JMIR Form Res 2022;6(6):e36176 (doi: 10.2196/36176; PMID: 35526139)

Multimedia Appendix 1: Descriptive statistics of laboratory measurements used in this study.

Laboratory measurements					
**Analysis (unit)**	**Median**	**IQR**	**Min**	**Max**	**number of measurements**
ALAT (U/L)	32	19 - 68	0	13526	102,745
alk. phosphatase (U/L)	100	68 - 174	7	6440	63,575
anorg. phosphate (mmol/L)	1.03	0.82 - 1.28	0.05	6.36	117,253
ASAT (U/L)	33	21 - 69	0	53100	84,288
base excess BE (mmol/L)	0.4	-2.7 - 3.4	-29.9	29.7	197,200
basophiles (10^9/L)	0.02	0.01 - 0.05	0	4.2	57,991
bicarbonate act. (mmol/L)	24.2	21.3 - 27.2	1.1	65.6	197,712
bilirubin direct (µmol/L)	8	4 - 31	0	1395	11,363
bilirubin total (µmol/L)	10	6 - 24	0	1424	63,609
body temperature (°C)	37	36.6 - 37.3	32	41.7	241,692
CA ion. at current pH (mmol/L)	1.17	1.12 - 1.22	0.11	6.13	206,924
CA ionized (pH 7.4) (mmol/L)	1.18	1.13 - 1.23	0.55	3.38	16,810
calcium total (mmol/L)	2.19	2.06 - 2.31	0.67	5.42	88,103
chloride (mmol/L)	107	104 - 110	65	138	212,535
C-reactive protein (mg/L)	45	11 - 109	0	747	234,293
creatinine (P) (µmol/L)	89	65 - 137	2	2063	269,901
creatinine ABL (µmol/L)	88	67 - 127	11	1498	18,071
eGFR by CKD-EPI (mL/min)	70	41 - 95	2	462	268,694
eosinophils (10^9/L)	0.08	0.01 - 0.21	0	44.3	58,000
erythrocytes (10^12/L)	3.33	2.88 - 3.96	0	9.43	353,108
Ferritin ECLIA (µg/L)	274	95 – 770	4	587880	6,587
g-glutamyltransferase (U/L)	95	38 - 249	3	5360	72,879
glucose (mmol/L)	8	6.39 - 10.3	0	152	986,700
hematocrit (L/L)	0.3	0.26 - 0.35	0	0.9	352,845
hemoglobin (g/L)	98	86 - 117	0	286	352,788
HbA1c (DCCT/NGSP) (%)	6.6	5.9 - 7.6	3.4	21.3	13,613
HbA1c (IFCC) (mmol/mol)	49	41 - 60	14	209	13,610
immature granulocytes (10^9/L)	0.07	0.03 - 0.17	0	25.49	26,974
iron (µmol/L)	8.6	5.4 - 13.5	1	148.8	3,884
leucocytes (10^9/L)	8.9	6.4 - 12.2	0	597.4	353,124
lymphocytes (10^9/L)	1.08	0.63 - 1.67	0	573.5	58,609
MCH (pg)	30	28 - 31	13	49	352,592
MCHC (g/L)	337	327 - 346	235	505	352,922
MCV (fL)	88	85 - 92	49	140	352,859
monocytes (10^9/L)	0.64	0.39 - 0.94	0	150.1	58,011
MPV (fL)	10.3	9.6 - 11	6.5	18.3	206,962
MPV (avg. thrombocyte-volume) (fL)	9.4	8.3 - 10.5	1.4	26.1	139,902
neutrophils (10^9/L)	6.76	4.15 - 10.37	0	95.74	58,742
normoblasts (/100 leucocytes)	1	0.5 - 3.5	0	100	3,266
normoblasts automated (/100 leuc.)	0	0 - 0.1	0	100	180,883
osmolality (mosm/kg)	291	279 - 303	210	429	10,212
pCO2 (mm Hg)	39	34 - 44	4	221	244,209
PCT hs (µg/L)	0.44	0.16 - 1.97	0.04	186.1	2,011

Analysis (unit)	Median	IQR	Min	Max	number of measurements
pH	7.411	7.359 - 7.454	6.8	7.798	249,795
pO2 (mm Hg)	77	54 - 101	1	595	242,142
potassium (mmol/L)	4.1	3.8 - 4.5	1.3	9	498,870
RDW (%)	14.7	13.5 - 16.4	10.6	43.5	210,406
RDW (erythrocyte-size variance) (%)	15.2	13.9 - 16.9	10.3	36.4	142,130
round epithelial cells (/µL)	3	1 - 8.1	0	924.6	13,085
sodium (mmol/L)	139	136 - 142	97	177	483,414
thrombocytes (10^9/L)	208	133 - 294	0	4380	353,115
transferrin (g/L)	1.78	1.39 - 2.23	0.27	4.73	3,285
