# Supplementary material for: Machine Learning Prediction of Hypoglycemia and Hyperglycemia From Electronic Health Records: Algorithm Development and Validation
Source: JMIR Form Res. 2022 Jul 18;6(7):e36176. doi: 10.2196/36176 (PMC9345028; doi:10.2196/36176)
Supplement: Multimedia Appendix 2 [file formative_v6i7e36176_app2.pdf]

Multimedia Appendix 2
Witte *et al.*, 2022: JMIR Form Res 2022;6(6):e36176 (doi: 10.2196/36176; PMID: 35526139)

Multimedia Appendix 2: List of variables used for model setup.

Variable	Type (values)	Rationale for variable use
**Patient information**		
age	integer	patient attributes may influence the susceptibility for decompensation
weight	numeric	
height	numeric	
administrative sex	factor (m, f)	
language	factor	language barrier may influence treatment in hospital
civil status	factor	civil status may have an influence on lifestyle, nutrition etc. and thus indirectly the susceptibility for decompensation
stay in intensive care unit	binary (0 = no, 1 = yes)	measure for overall severity of case (with dysglycemia as frequent consequence of severe disease)
**Diagnosis history**		
diagnosis information for twenty ICD-10-codes listed in the inclusion criteria (E10, E11, …, R81), one variable per code	binary (0 = not diagnosed previously, 1 = diagnosed previously)	diagnoses of diabetes and / or comorbidities may increase risk of decompensation
sum of all diabetes(-related) diagnoses in the patient’s past (variable increase of 1 per positive diagnosis)	integer (e.g. diagnoses of E11, E66 and K85 => feature value of 3)	
binary indicator variable for one or more of the ICD-10-codes listed in the inclusion criteria in the patient’s past	binary (0 if no diagnoses, 1 otherwise, e.g. E11, E66 and K85 => feature value of 1))	
binary indicator for one or more of the main ICD-10-codes for diabetes (E10 through E16) in the patient’s past	binary (0 = no, 1 = yes, e.g. E10 => 1, E11 and E14 => 1))	
decompensation during previous hospitalizations	binary (0 = no, 1 = yes)	knowledge about previous decompensation may reduce risk
number of previous hospitalizations of patient	integer	measure for severity of case
**Medication (antidiabetic drugs ADD)**		
History of anti-diabetic drug (ADD) prescription Have ADDS been administered in the past (before the current case)?	binary (0 = no, 1 = yes)	Patients know to have received ADDs may be more or less susceptible to decompensation
Anti-diabetic drug (ADD) administration within current case, time window before (!) decompensation event (or patient discharge)	binary (0 = no, 1 = yes)	potential measure for severity of case or risk of decompensation
**Laboratory information**		
total number of laboratory analyses carried out during stay	integer	indirect measure for severity of case
total number of laboratory analyses carried out during stay, normalized to length of stay or time until decompensation	integer	indirect measure for severity of case
number of extreme measurements flagged as unlikely (by outlier detection)	integer	indirect measure for severity of case
time of last measurement before decompensation or discharge in hours	integer	The longer a patient may remain non-decompensated the less likely it may become to happen in the clinical context

decompensation of opposite type has happened before within case	binary (0 = no, 1 = yes)	If a patient has suffered from hyperglycemia within the case, he might be more susceptible for hypoglycemia and vice versa
**Features extracted from laboratory analysis history of current case (before decompensation or patient discharge, for each analyte)**		
mean	numeric	average measure of analyte value (intentionally, the mean is favored over the median as it is less robust to outliers)
standard deviation	numeric	measure of fluctuation of values
analysis count	integer	measure for severity of case and importance of analyte
interquartile range (IQR)	numeric	robust measure of fluctuation of values
value of last measurement (before decompensation or final measurement)	numeric	“current” status
value deviating most from analysis median of whole patient population	numeric	most extreme value of analyte within case
data range (difference of minimum and maximum)	numeric	measure for range of values
data trend between second-last and last measurement before decompensation or patient discharge	numeric	trend of values (up / down, strong increase/decrease or rather constant)
